# The Lack of Cytotoxic Effect and Radioadaptive Response in Splenocytes of Mice Exposed to Low Level Internal β-Particle Irradiation through Tritiated Drinking Water *in Vivo*

**DOI:** 10.3390/ijms141223791

**Published:** 2013-12-05

**Authors:** Matthew Flegal, Melinda Blimkie, Sandrine Roch-Lefevre, Eric Gregoire, Dmitry Klokov

**Affiliations:** 1 Chalk River Laboratories, Atomic Energy of Canada Limited, Chalk River, ON K0J1P0, Canada; E-Mails: flegalm@aecl.ca (M.F.); blimkiesm@aecl.ca (M.B.); 2 Institute of Radioprotection and Nuclear Safety, B.P. 17, Fontenay aux Roses Cedex 92262, France; E-Mails: sandrine.roch-lefevre@irsn.fr (S.R.-L.); eric.gregoire@irsn.fr (E.G.)

**Keywords:** β-radiation, tritium, cytotoxicity, radioadaptive response, apoptosis, *in vivo*

## Abstract

Health effects of tritium, a β-emitter and a by-product of the nuclear industry, is a subject of significant controversy. This mouse *in vivo* study was undertaken to monitor biological effects of low level tritium exposure. Mice were exposed to tritiated drinking water (HTO) at 10 KBq/L, 1 MBq/L and 20 MBq/L concentrations for one month. The treatment did not result in a significant increase of apoptosis in splenocytes. To examine if this low level tritium exposure alters radiosensitivity, the extracted splenocytes were challenged *in vitro* with 2 Gy γ-radiation, and apoptotic responses at 1 and 24 h were measured. No alterations in the radiosensitivity were detected in cells from mice exposed to tritium compared to sham-treated mice. In contrast, low dose γ-irradiation at 20 or 100 mGy, resulted in a significant increase in resistance to apoptotic cell death after 2 Gy irradiation; an indication of the radioadaptive response. Overall, our data suggest that low concentrations of tritium given to mice as HTO in drinking water do not exert cytotoxic effect in splenocytes, nor do they change cellular sensitivity to additional high dose γ-radiation. The latter may be considered as the lack of a radioadaptive response, typically observed after low dose γ-irradiation.

## 1. Introduction

Various types of ionizing radiation vary in their potency to induce damage to cells and tissues depending on their linear energy transfer (LET). High LET radiations (α-, protons, heavy ions) produce more damage per dose unit compared to low LET radiations (γ-, X-ray, β-emitters). However, even among low LET radiations, there may be differences in the extent and type of biological consequences. Thus, soft X-rays are known to be more damaging compared to ^60^Co γ-rays [1]. Relative biological effectiveness (RBE) is a factor introduced by radiation protection authorities to account for those differences among different radiation types [2].

Tritium, a radioactive isotope of hydrogen, is an internal β-particle emitter. Its potential toxicity and human health effects represent a major concern since it is produced by nuclear power reactors and can readily enter environmental routes in the form of tritiated water (HTO) or organically bound tritium (OBT). In spite of a large number of published experimental data on biological effects of tritium, there is no consensus on how to regulate exposure to tritium for the public. For instance, the guidelines for tritium in drinking water range from 100 Bq/L in Europe [3] to 75,000 Bq/L in Australia [4]. Experimental or epidemiological evaluations of RBE for tritium vary from 1 to 12 [5]. There are numerous contributors to such discrepancies; however, the most significant ones are the use of inappropriate reference radiation types and irradiation modes (acute or chronic) and inappropriately high dose and dose-rates (reviewed in [5]). This ambiguous situation leaves room for public inquiries [6,7], scientific debates [8–10], and may stir up unnecessary public concern. Obviously, there is a need for radiobiological studies examining biological responses in mammalian model organisms to low tritium exposure levels over extended periods of time.

Therefore, this study was designed to evaluate potential cytotoxic effects of tritium given to mice *in vivo* in the form of HTO in drinking water at very low concentrations. We chose three exposure levels, with the lowest one being below or slightly above regulatory limits in some countries. However, even the highest tritium concentration used in this work was significantly lower compared to those used in the vast majority of published reports. Also we used a chronic exposure mode via drinking water which is relevant to most occupational and environmental exposure scenarios. Furthermore, the use of additional challenging high dose γ-irradiation of cells from tritium exposed animals allowed us to evaluate any changes in cellular radiosensitivity that would have otherwise been undetectable.

## 2. Results and Discussion

### 2.1. The Lack of Apoptosis Due to Tritium Exposure

**Figure 1.**
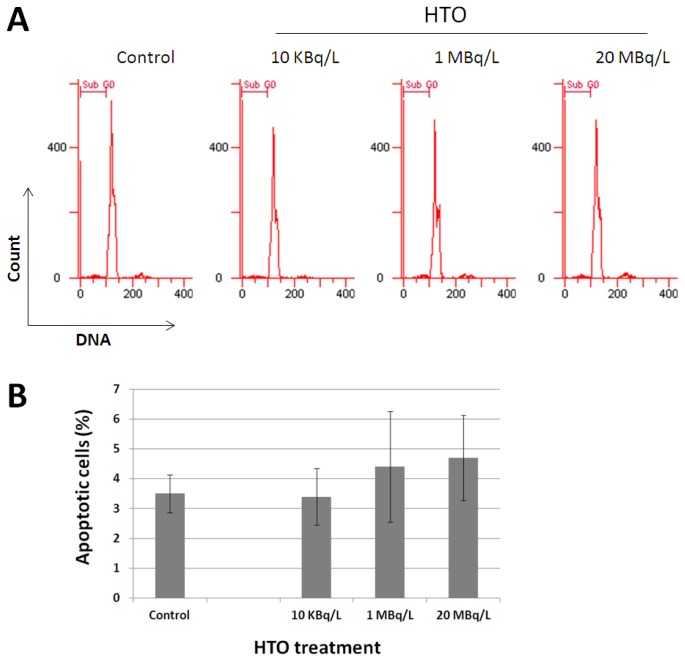
Exposure to low tritiated drinking water (HTO) concentrations does not cause cytotoxicity *in vivo*. (**A**) Representative DNA histograms of mouse splenocytes obtained by flow cytometry. Mice were sham- or HTO-treated at indicated concentrations for one month. Splenocytes were extracted, fixed and stained with Propidium Iodide (PI) for flow cytometric analysis of cell cycle distribution. SubG0 region containing apoptotic cells was used for quantification; (**B**) Quantification of apoptosis in splenocytes from the control and HTO-treated mice. Mean values ± SD are shown (*n* = 5/group).

We used flow cytometry to examine the rate of apoptosis by measuring the SubG0/G1 cell fraction. Representative histograms of DNA content in splenocytes obtained from the control and tritium exposed mice are shown in Figure 1A. The results of quantification are presented in Figure 1B. The rate of apoptosis in the control group was approximately 3%. One-month exposure to HTO in drinking water did not lead to a statistically significant increase in the fraction of apoptotic cells (Figure 1B). Although a slight increase was observed in cells from 1 and 20 MBq/L groups, it was not statistically significant (*p* = 0.35 and 0.18 (*n* = 5/group) for 1 and 20 MBq/L groups, respectively).

The lack of cytotoxicity in our experiments is not surprising when total accumulated doses from the tritium exposure are considered. It was evaluated that 10 KBq/L, 1 MBq/L and 20 MBq/L HTO exposures for 1 month would result in the absorbed doses of 0.0096, 0.96 and 20.8 mGy, respectively. This evaluation was based on the results of our preliminary experiments examining the kinetics of tritium accumulation and retention in mouse tissues using the measurement of tritium levels by a tissue oxidizer and showing that the body steady state level of tritium was reached in 14 days and was stable for the rest of the exposure at about 40% of the treatment concentration (data not shown). Although lymphoid cells, that splenocytes belong to, are known for high radiosensitivity, our results indicate that chronic exposure to low doses of β-radiation from HTO in drinking water cause no cytotoxicity in splenocytes.

### 2.2. Radiosensitivity of Splenocytes from Tritium-Exposed Mice Is Not Altered

**Figure 2.**
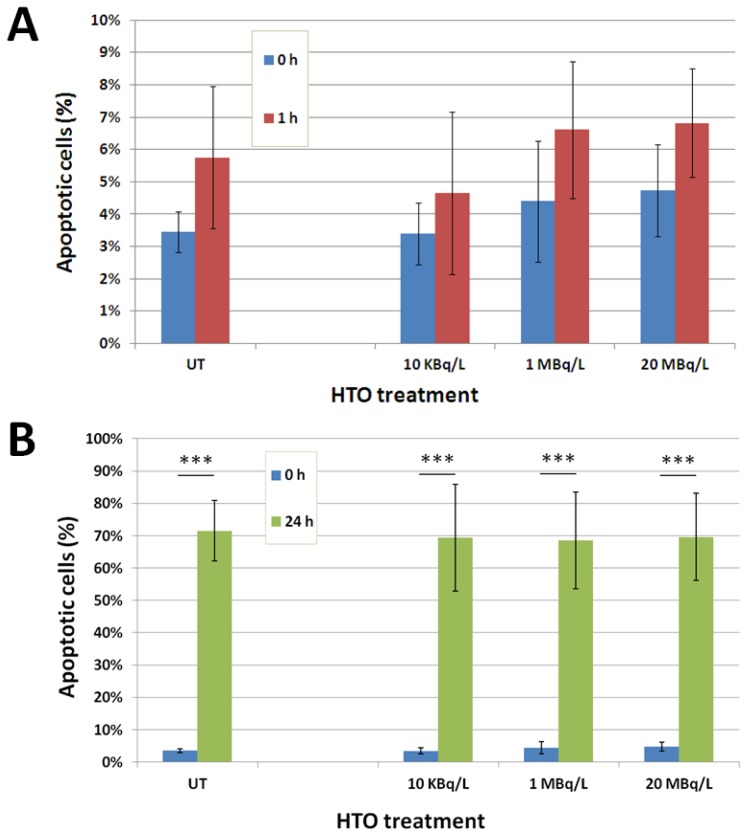
Exposure to low HTO concentrations does not alter cellular radiosensitivity. Apoptosis (SubG0 cell population) was measured in splenocytes isolated from the indicated treatment groups (*t* = 0 h means before the challenging irradiation; untreated (UT) is control; blue bars) and challenged *ex vivo* with 2 Gy γ-irradiation. Then cells were incubated in RPMI (Roswell Park Memorial Institute) medium for 1 h (**A**) or 24 h (**B**). Cells were then fixed and processed for flow cytometry as described in Experimental Section. Mean values ± SD are shown (*n* = 5/group). *** denotes statistically significant difference compared to control at *p* < 0.001.

To undergo apoptotic cell death, cells must sustain a significant amount of damage. Although we did not see abnormal apoptosis rates due to tritium exposures in our experiments, it was reasonable to hypothesize that the low level of damage inflicted by the β-exposure, even though not enough for triggering cell death responses, may still be able to cause subtle molecular changes that could potentially alter cellular stress resistance or radiosensitivity. To examine this hypothesis, we irradiated splenocytes isolated from control or tritium-exposed mice to a high dose of γ-radiation of 2 Gy. Then, we measured early (1 h) and late (24 h) apoptotic responses. Although the apoptotic responses we examined develop in the *ex vivo* context, our previous results had indicated that double strand break responses facilitated by γH2AX in splenocytes are similar between *ex vivo* and *in vivo* conditions during first 24 h after the challenging irradiation [11]. It allowed us to believe that similar apoptotic responses are expected *ex vivo vs. in vivo*. As expected, we saw no significant apoptotic response in control cells at 1 h after 2 Gy irradiation (Figure 2B). Indeed, under normal conditions apoptosis starts at 4–6 h after the infliction of damage [12]. Apparently, this was not changed due to the tritium exposure, as the rate of apoptosis in the tritium exposed groups at 1 h after the challenging exposure was not higher than that before the challenge (Figure 2A).

Similarly, we saw no difference in the level of apoptosis between control and tritium groups at 24 h after the 2 Gy γ-irradiation (Figure 2B). This result indicates that exposure to very low doses of internal β-particles from HTO does not affect cellular radiosensitivity towards higher damaging radiation doses. Interestingly, β-radiation from tritiated thymidine was shown to render cultured human lymphocytes more resistance to high dose γ-radiation, a phenomenon called adaptive response [13]. However, doses used by the authors were substantially higher that the doses used in our study and the exposure times significantly shorter. γ-radiation at low doses has since been widely used to induce radiation adaptive responses in a variety of experimental models and endpoints [14].

### 2.3. Low Dose γ-Radiation Induces the Adaptive Response in Mouse Splenocytes

**Figure 3.**
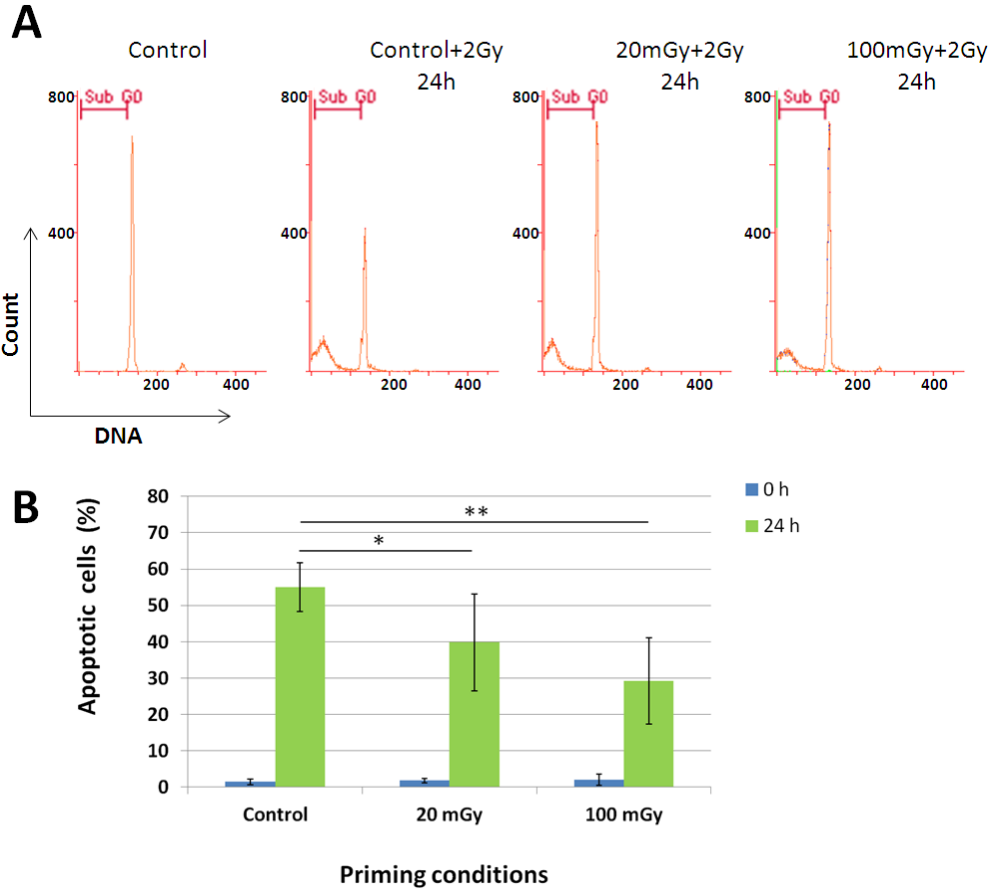
Exposure of mice to low doses of γ-radiation *in vivo* induces resistance of splenocytes to subsequent high dose γ-irradiation. (**A**) Representative DNA histograms of mouse splenocytes obtained by flow cytometry. Mice were sham-treated or irradiated with 20 or 100 mGy. Twenty four hours later isolated splenocytes were challenged with 2 Gy γ-radiation and incubated for 24 h. Apoptosis then was evaluated and quantified (**B**) using flow cytometry as a SubG0 cell population. Mean values ± SD are shown (*n* = 5/group). * and ** denotes statistically significant difference compared to control at *p* < 0.05 and *p* < 0.01, respectively.

We then examined whether low dose γ-radiation could induce the radioadaptive response using the apoptosis endpoint in our experimental mouse model. We exposed animals to whole body γ-irradiation with doses of 20 or 100 mGy; the control group was sham-irradiated. Only the lower adapting γ-dose overlapped with the doses accumulated as a result of tritium exposure in our experiments; the higher adapting dose of 100 mGy was still used given our previous data showing its ability to trigger radioadaptive responses [15]. Twenty four hours later, the extracted splenocytes were irradiated with a challenging dose of 2 Gy. The apoptotic responses were measured by flow cytometry 24 h after the challenging irradiation (Figure 3A). We observed lower apoptosis rates in cells from low dose irradiated mice compared to the control (Figure 3B). The higher dose of 100 mGy triggered higher protection from apoptosis (29% *vs.* 55% apoptotic cells in the control group, *p* = 0.007), whereas milder protection was seen with a 20 mGy dose (40% *vs.* 55% in the control, *p* = 0.02). Obviously, the γ-radiation doses used for the radioadaptive response induction are significantly higher than those accumulated from the tritium exposure. However, this result should be regarded as a proof of principle that low dose exposures can indeed affect radiosensitivity of splenocytes in our model. Therefore, the lack of this effect in case of low dose tritium exposure provides evidence that no cellular or molecular pathways leading to either radiosensitization or radioresintance are affected by the β-exposure.

Aiming to use occupationally and environmentally relevant concentrations of tritium in our study, we expected that the likelihood of detecting any deleterious effects was low. Indeed, Ijiri reported that 3.7 MBq/mouse of injected HTO had no effect on cell death in the small intestine or the descending colon [12]. The exposure mode and concentrations used in our study would be equivalent to around 100 KBq/mouse for the highest dose group of 20 MBq/L. In another study, thymus weight loss, which represents the results of thymocytes killing and is an indirect measure of apoptosis in cells that are very similar to the splenocytes used in our study, was monitored in rats receiving injections of HTO [16]. The authors showed that the lowest HTO dose used, 1.1 MBq/g (compare to our 10 KBq/g for the highest dose group) had little effect on thymus weight loss.

It is worth pointing out that OBT may exert different cellular responses, since tritium in this case may be incorporated into DNA precursors, such as thymidine, glycine, *etc.*, and induce localized heavy damage to DNA strands [17]. In addition, other endpoints, more relevant to radiological risks, evaluated mostly for cancer, should be considered for the evaluation of RBE of tritium exposures [18–20]. Our future work will, therefore, focus on other endpoints, such as molecular damage to DNA (DNA double strand breaks), chromosomal aberrations, gene expression changes, epigenetic alterations, and will examine effects from longer exposures to both HTO and OBT.

## 3. Experimental Section

### 3.1. Reagents

RPMI medium was purchased from HyClone (Fisher Scientific, Toronto, ON, Canada). Ethanol was from Commercial Alcohols, Inc (Brampton, ON, Canada). Flow cytometry supplies, including Flow Check Fluorospheres, were obtained from Beckman Coulter Canada, Inc (Mississauga, ON, Canada). Propidium Iodide, Triton X-100, Tween-20 were purchased from Sigma-Aldrich St. Louis, MO, USA. DAPI (4′,6-diamidino-2-phenylindole dihydrochloride hydrate) was purchased from Invitrogen (Burlington, ON, Canada). Bovine serum albumin was obtained from VWR, Mississauga, ON, Canada and foetal bovine serum (FBS) was purchased from Gibco (Invitrogen, Burlington, ON, Canada).

### 3.2. Mice Housing and Care

Adult male or female C57BL/6J mice (Jackson laboratory, Bar Harbor, ME, USA) aged 7–8 weeks were used in this study. The animals were acclimatized for a minimum of one week after their arrival and then randomly assigned to experimental groups (5 mice per group). All animals were housed in a specific-pathogen-free environment in the Biological Research Facility at Chalk River Nuclear Laboratories (Chalk River, ON, Canada). Mice were maintained in cages inside ventilated cage racks equipped with an automatic ventilation and watering system. In tritium exposure experiments, drinking water with tritium was provided from bottles *ad libitum*. Animals were fed *ad libitum* and their health status was examined daily. The facility was equipped with automatic computer-controlled temperature (23 °C), air ventilation and 12-h light/dark cycle. Tests for infections were performed routinely and all mice tested were negative. All protocols were performed in accordance with the guidelines of the Canadian Council on Animal Care [21] with the approval of the local Animal Care Committee.

### 3.3. Tritium Exposure

HTO stock with the activity of 3.7 × 10^9^ KBq/L was obtained locally from National Research Universal reactor facility at Chalk River Laboratories (Chalk River). The HTO stock was diluted in animal drinking water to obtain 10 KBq/L, 1 MBq/L and 20 MBq/L working concentrations. The resulting activities were measured using Tri-Carb 1900 liquid scintillation counter (Packard Instruments, Downer’s Grove, IL, USA) and confirmed to be within 3% of the target concentration. Animals were given the treatment water via bottles *ad libitum*. The water was changed once during the 1-month exposure period, two weeks after the start of exposure. One-month exposure was in fact four calendar weeks or 28 days. Male mice were used in the tritium exposure experiment. Control mice were maintained in a rack with negative air pressure to avoid tritium contamination through breathing air. Negative pressure was maintained in racks with animals exposed to tritium.

### 3.4. Low Dose and Challenging γ-Irradiation

For low dose γ-irradiation, mice in plastic cages were irradiated with low doses of 20 or 100 mGy using an open beam ^60^Co-γ-source (γBeam-150, Atomic Energy of Canada Limited, Chalk River) at a dose rate of 1 mGy/min. Control mice were sham-irradiated. Twenty three hours after low dose *in vivo* γ-irradiation or after 1-month HTO exposure, the mice were sacrificed by cervical dislocation and spleens were removed and rinsed in phosphate buffered saline. Splenocytes were isolated and cultured in RPMI media supplemented with 10% FBS, 2 mM l-glutamine in T25 flasks at 37 °C and a 5% CO_2_ and 95% air atmosphere. Twenty four hours after low dose γ-irradiation of mice (approximately 30 min after culture initiation in a CO_2_-incubator), flasks with cell suspensions were irradiated with a challenging dose of 2 Gy using a GammaCell-220 device (Atomic Energy Canada Limited, Chalk River) at a dose rate of 6.6 Gy/min (^60^Co γ-ray source) at room temperature. Immediately after irradiation the cell cultures were returned to the CO_2_-incubator and incubated for various times before sampling. At time points 0 (without 2 Gy dose), 1 and 24 h after challenging irradiation, cell aliquots were collected and processed for flow cytometry.

### 3.5. Flow Cytometry

Mouse splenocytes (10^6^ cells) were fixed in 1 mL of 70% ethanol/30% Tris-buffered saline (TBS) at various times after a challenging dose of 2 Gy and stored at −20 °C. The fixed cells were stored for up to 3 months until all samples were collected and ready for flow cytometry analysis of samples in one day. On the day of analysis, 1 mL of cold 1% FBS/TBS was added to 1 mL of fixed sample and cells were centrifuged and resuspended in 1 mL of cold TST (Tris buffered Saline-Triton) buffer (4% FBS, 0.1% TritonX-100 in TBS). Cells were incubated for 20 min on ice for rehydration and then centrifuged and resuspended in TBS. Cells were centrifuged again and resuspended in 400 μL of PBS containing 50 μg/mL Propidium Iodide. Samples were analyzed using the Cell Lab Quanta SC flow cytometer (Beckman Coulter Inc., Miami, FL, USA) with an MPL (Multi-Platform Loader) auto-sampler for a 96-well plate format. For quality control, verification of the flow cytometer’s optical alignment and fluidics system using Flow-Check Fluorospheres (Beckman Coulter Inc.) was run every time before cell samples. PI (DNA content) fluorescence were triggered by a 488 nm argon laser and detected and measured in the FL3 (670LP) channel. Readings from 10,000 cells were collected and analyzed by Quanta Analysis software (Beckman Coulter Inc.). SubG0/G1 cell populations representing apoptotic cells were quantified.

### 3.6. Statistical Analysis

The mean values for apoptotic cell populations were calculated from 5 mice per treatment group. Standard deviations were used to evaluate errors. Treatment groups were compared using two-tailed Student’s *t*-test. A significance threshold was set up to *p* < 0.05.

## 4. Conclusions

In this study, we evaluated toxicity resulting from the prolonged exposure of mice to low level tritium in the drinking water. We failed to observe an increased level of apoptosis in splenocytes from the HTO-treated mice compared to the sham-treated animals, suggesting the lack of adverse health effects. Furthermore, the HTO treatment did not enhance cellular radiosensitivity towards high dose γ-radiation, nor did it result in the radioprotection normally seen after low dose γ-irradiation and referred to as a radioadaptive response. While our future studies will focus on a variety of other end points, such as DNA double strand breaks, chromosomal rearrangements, and genetic and epigenetic alterations, the presented data provide the first indication of the lack of low level tritium cytotoxicity *in vivo*.
